# Anemia Management in Peritoneal Dialysis: Perspectives From the Asia Pacific Region

**DOI:** 10.1016/j.xkme.2021.01.011

**Published:** 2021-04-20

**Authors:** Philip Kam Tao Li, Agnes Shin Man Choy, Sunita Bavanandan, Wei Chen, Marjorie Foo, Talerngsak Kanjanabuch, Yong-Lim Kim, Masaaki Nakayama, Xueqing Yu

**Affiliations:** 1 Department of Medicine and Therapeutics, Carol and Richard Yu Peritoneal Dialysis Research Centre, Prince of Wales Hospital, The Chinese University of Hong Kong, Shatin, Hong Kong, SAR, China; 2 Department of Nephrology, Hospital Kuala Lumpur, Kuala Lumpur, Malaysia; 3 Department of Nephrology, The First Affiliated Hospital, Sun Yat-sen University, Key Laboratory of Nephrology, National Health Commission of China and Guangdong Province, Guangzhou, China; 4 Department of Renal Medicine, Singapore General Hospital, Singapore; 5 Center of Excellence in Kidney Metabolic Disorders and Division of Nephrology, Department of Medicine, Faculty of Medicine, Chulalongkorn University, Bangkok, Thailand; 6 School of Medicine, Kyungpook National University, Daegu, South Korea; 7 Kidney Center, St. Luke’s International Hospital, Tokyo, Japan; 8 Department of Nephrology, Guangdong Provincial People’s Hospital, Guangzhou, China; 9 Guangdong Academy of Medical Sciences, Guangzhou, China

**Keywords:** Anemia, peritoneal dialysis, Asia Pacific

## Abstract

Anemia is an important complication in patients with chronic kidney disease. Peritoneal dialysis (PD) is one of the most common modalities of kidney replacement therapy for patients with end-stage kidney disease. PD is particularly prevalent in the Asian Pacific region. Among the different countries and regions, including mainland China, Hong Kong, Japan, Malaysia, Singapore, South Korea, and Thailand, PD accounts for 2.8% to 74.6% of the dialysis population. In addition, 82% to 96% of the PD populations from these countries and regions are receiving erythropoiesis-stimulating agents (ESAs). Asian Pacific countries and regions follow the latest KDIGO (Kidney Disease: Improving Global Outcomes) guidelines for the initiation of treatment of anemia in PD patients. The types of ESAs commonly used include shorter-acting (epoetin alfa and beta) and longer-acting agents, including darbepoetin alfa or methoxy polyethylene glycol-epoetin beta. The most commonly used ESAs in Mainland China, Malaysia, Singapore, and Thailand are the shorter-acting agents, whereas in Hong Kong, Japan, and South Korea, longer-acting ESAs are most common. Oral iron therapy is still the most commonly used iron supplement. The route and dosage of iron administration in PD patients requires more research studies. With the introduction of oral hypoxia-inducible factor prolyl hydroxylase inhibitors into clinical use, the landscape of treatment of anemia in the PD population in the Asia Pacific region may change in the coming years.

## Introduction

Anemia remains an important complication in patients with chronic kidney disease (CKD). As kidney disease progresses, anemia increases in prevalence and affects nearly all patients with stage 5 CKD and is a main cause of morbidity and mortality.^1^ Anemia begins in early stages of CKD and can worsen and become difficult to manage with decreasing kidney function.^2^

Anemia is defined as a low level of hemoglobin (Hb) < 12 g/dL in women and <13 g/dL in men among patients with CKD.^3^ In patients with CKD, there is an absolute or a relative deficiency of erythropoietin production, which results in the development of anemia. Other factors that can also contribute to anemia in this population include iron deficiency, inflammation, hemolysis, blood loss, and nutritional deficiency.^4^ In addition to the decrease in erythrocyte production, patients with CKD also have shortened survival of red blood cells.^5^ These 2 factors result in reduced oxygen delivery to the body’s organs and tissues, leading to symptoms of anemia. which is associated with reduced quality of life, increased cardiovascular diseases, hospitalizations, cognitive impairment, and mortality.^1^

Since the landmark study of the use of erythropoietin in 1987, the cornerstone of anemia treatment in CKD has been erythropoiesis-stimulating agent (ESA) therapy.^6^ The KDIGO (Kidney Disease: Improving Global Outcomes) guidelines recommend that patients with stage 5 CKD receiving dialysis should start ESA therapy when their Hb level is between 9.0 and 10.0 g/dL to prevent the concentration from decreasing to <9.0 g/dL.^3^ It is also suggested that ESAs should not be used to maintain Hb concentrations at >11.5 g/dL in adult patients with CKD.^3^

Asia is the largest and most densely populated continent. Of a world population of 6.915 billion, Asia constitutes 4.165 billion.^7^ Both the prevalence and incidence of end-stage kidney disease (ESKD) have significantly increased in many Asian countries and regions in recent years.^8^^,^^9^ There is a particularly high prevalence of peritoneal dialysis (PD) for treating patients with ESKD in Asia compared with the rest of the world.^10^

A roundtable discussion among nephrologists and opinion leaders from mainland China, Hong Kong, Japan, Malaysia, Singapore, South Korea, and Thailand on the management of anemia in PD patients in the Asia Pacific region was organized in November 2019. The roundtable set out to describe the current status of anemia in PD patients and the management issues in relation to ESA and iron therapy, with the goal of improving the current management of anemia in the Asia Pacific region. This report summarizes the discussion findings.

## Treatment of Anemia in CKD

### ESA Treatment

ESAs are the hallmark therapy for treating anemia in CKD.^6^ The first available agent was epoetin alfa, a shorter-acting ESA, with a half-life of 6.8 hours for intravenous (IV) administration.^6^ Another commonly used shorter-acting erythropoietin is epoetin beta. Other ESAs developed include longer-acting agents such as darbepoetin alfa, with a half-life approximately 2 to 3 times longer than epoetin alfa, and methoxy polyethylene glycol-epoetin beta, which has a significantly increased serum half-life of 130 hours with IV administration.^4^

PD patients seem to experience less anemia than hemodialysis (HD) patients. In a retrospective analysis of Medicare patients in the United States involving 17,842 PD and 256,942 HD patients in 2011, a total of 71.4% of PD patients received ESAs in 2011 compared with 86.9% of HD patients.^11^ Anemia seems to be less severe in PD compared with HD. This may be because HD patients generally have less residual kidney function, are prescribed heparin more often, and have more long-term blood loss. In addition, HD patients generally experience more volume overload and inflammation and have more comorbid conditions that can require transfusions.^11^

Iron deficiency is also more common in patients treated by HD.^12^ Iron losses in stable HD patients are approximately 2.0 to 2.5 g per year and appear to be mostly a result of blood loss and destruction by the dialysis circuit, as well as hospitalizations and procedures that may involve blood loss. PD patients also experience iron deficiency, at approximately 1 g per year, which is lower than HD patients and is mostly due to gastrointestinal blood loss, diagnostic phlebotomy, and other medical procedures.^12^

### Hypoxia-Inducible Factor Inhibitor

Tissue hypoxia is sensed by the body through the hypoxia-inducible factor (HIF) system, leading to recognition of anemia. Central to this function are 2 proteins, HIF-α and HIF-β. In normoxic conditions, HIF-α is hydroxylated by prolyl hydroxylase domain proteins and undergoes proteosomal degradation. When tissue hypoxia occurs, instead of undergoing degradation, HIF-α translocates to the nucleus and binds with HIF-β. This process activates a hypoxia response element and leads to the gene transcription of erythropoietin.^4^ It has been shown in clinical trials that HIF stabilizers can effectively increase Hb levels of patients with CKD, both those receiving dialysis and those not receiving dialysis, without any serious adverse effects. HIF stabilizers restore production of erythropoietin and also improve iron metabolism by reducing hepcidin levels.^13^

There are at least 5 HIF prolyl hydroxylase inhibitors (HIF-PHIs) currently being evaluated in clinical trials: roxadustat, daprodustat, vadadustat, molidustat, and enarodustat.^13^ At present, roxadustat, vadadustat, and daprodustat are commercially available HIF-PHIs on the market for clinical use. In a recent large-scale phase 3 clinical trial in China, it was shown that oral roxadustat was noninferior to parenteral epoetin alfa as therapy for anemia in Chinese patients undergoing dialysis.^14^ In another recent Japanese study, roxadustat maintained Hb levels within 10 to 12 g/dL in patients receiving HD and was noninferior to darbepoetin alfa.^15^ Another study showed that oral daprodustat was noninferior to darbepoetin alfa as measured by mean Hb levels in Japanese patients receiving HD switched from ESAs.^16^

### Iron Treatment

Before starting ESA treatment, iron status should be assessed. Other possible causes of anemia should be excluded, including occult bleeding, hemolysis, and vitamin B_12_ or folic acid deficiency. Iron supplementation has been demonstrated to improve the erythropoietic response to ESA treatment.^17^^,^^18^ Iron treatment can be administered either in oral or IV form. Oral iron effectiveness may be reduced by a number of factors, including possible gastrointestinal side effects, frequent administration, and a reduction in enteral absorption due to its interaction with food, phosphate binders, and reduced gastric acidity.^12^ For IV iron, iron dextran, iron sucrose, ferric gluconate, ferumoxytol, ferric carboxymaltose, and iron isomaltoside are common formulations used.^19^ IV iron can be given as bolus dosing and maintenance dosing in HD patients. Data suggest that maintenance IV iron is effective and associated with lower risk for infection compared with bolus dosing. In addition, when maintenance iron dosing was compared with no iron therapy, maintenance iron was not associated with additional risk.^20^

The recent Proactive IV Iron Therapy in Haemodialysis Patients (PIVOTAL) trial in HD patients found that a high-dose regimen of IV iron sucrose (400 mg monthly, unless ferritin concentration is >700 μg/L or transferrin saturation [TSAT] is ≥40%) when given proactively resulted in a significantly lower risk for death or major nonfatal cardiovascular events as compared with a reactive low-dose regimen (0-400 mg monthly, with iron administered if ferritin concentration was <200 μg/L or TSAT was <20%).^21^ The trial found that this method of dosing resulted in a significantly lower ESA dose and a lower blood transfusion incidence. In addition, the incidence of infection and hospitalization did not differ significantly between the 2 groups.

There have been very few randomized controlled trials using IV iron in PD patients.^22^ Singh et al^22^ randomly assigned 126 PD patients and found that IV iron sucrose is effective as an adjunct to ESA therapy in patients with anemia and CKD treated by long-term PD and can be safely administered as 300 mg over 1.5 hours or 400 mg over 2.5 hours. The Kidney Disease Outcome Quality Initiative guidelines do not recommend a preferred route of iron administration for PD patients.

A recent Cochrane database analysis found with a low certainty of evidence that IV iron increased Hb, ferritin, and transferrin levels in patients with CKD as compared with oral iron. IV iron also helped more patients achieve target Hb levels and reduced their need for ESAs as compared with oral iron.^23^ However, the review did not find sufficient evidence to determine whether the method of iron administration affects all-cause mortality, cardiovascular death, or quality of life because most of the studies had only short follow-up periods.^23^

An important concern is the high possible risk for iron overload in dialysis patients who are treated with IV iron at doses recommended by current anemia management guidelines. An overload of iron in the liver can lead to an increase in hepcidin production and elevated plasma levels, which in turn can activate macrophages of atherosclerotic plaques.^24^ A recent review observed that although serum ferritin concentration and TSAT can be used to help guide decision making about whether to prescribe IV iron therapy, it is still challenging to determine what the optimal iron status should be in individual patients with CKD, and further study is needed.^25^ New approaches to iron therapy and anemia management that incorporate innovative pharmacometric and economic methods are being designed to reduce potential side effects of excessive IV iron while at the same time keeping Hb levels stable and not requiring an increase in ESA dose.^24^

## Anemia Treatment in PD Patients in the Asia Pacific Region

Differences in treatment patterns are observed across different Asian countries, suggesting different clinical policies and practices. Nephrologists from different Asian regions vary in their choices of dialysis modality, Hb target, thresholds for ESA, type of ESA use, and iron prescription. This can be attributed to differences in government funding policy, cultural differences, and variation in adoption and interpretation of different sets of clinical guidelines.

### Mainland-China

According to the Chinese National Renal Data System 2018, the numbers of PD and HD patients are growing rapidly. The incident dialysis population of mainland China was 105,223 in 2017.^26^ There were 90,166 incident HD patients and 15,057 incident PD patients, respectively, in 2017. The total number of prevalent HD patients also increased from 248,016 in 2012 to 524,467 in 2017, treated at 5,479 HD centers. The total number of prevalent patients receiving PD increased from 37,942 in 2012 to 86,344 in 2017. The PD patient number further increased to 99,145 treated at 1,560 PD centers in 2018. Guangdong and Zhejiang provinces had the highest number of PD patients (>9,000) in mainland China in 2018.^26^

In 2016, the mean Hb level of PD patients in mainland China was 10 g/dL, and the overall percentage of target Hb levels of 10 g/dL achieved was 57.8%. A multicenter cohort study involving 9 centers from the largest dialysis facilities in 6 cities in mainland China showed that 89.1% of PD patients with anemia were treated with erythropoietin.^27^ Data from Sun Yat-sen University showed that in 2018, a total of 43% of PD patients required weekly ESA doses > 9,000 units while 28% required 6,000 to 9,000 units and 18% required 3,000 to 6,000 units, respectively.^28^ The ESAs used were the shorter-acting agents. In the Sun Yat-sen University experience, 99% of PD patients who received an iron supplement used oral formulations while 1% were given parenteral iron therapy.^28^

### Hong Kong

With the PD-first policy instituted in 1985, Hong Kong had the highest prevalence of patients receiving PD in the Asia Pacific region and the world.^10^ Under this policy, all patients who required dialysis therapy were first treated with PD unless they had medical contraindications to PD. Patients could choose HD therapy first based on personal preference, but they would be responsible for the costs.^29^^,^^30^ Hong Kong was the first region in the world to adopt a PD-first policy, and among all Asian countries with a registry, Hong Kong is the only place with more patients treated by PD than by HD. The ESKD burden in Hong Kong is reflected in the Hong Kong Renal Registry.^31^ The total number of dialysis patients increased from 4,189 to 6,094 from 2008 to 2018.^30^ Among all the dialysis patients in 2018, a total of 74.6% were receiving PD.

ESAs were introduced for kidney-related anemia, starting with self-payment in the 1980s, then subsidized programs, and finally through hospital provision. Currently, patients under Hospital Authority who are receiving dialysis with Hb levels < 10.0 g/dL are fully subsidized by the Hospital Authority for the use of ESA therapy.^32^ With this government funding, the percentage of dialysis patients receiving ESAs increased from 32.0% in 2005 to 65.0% in 2013.^32^ In 2017, a total of 71.3% of dialysis patients in Hong Kong were treated by ESAs, amounting to 4,467 patients. In 2018 in the Prince of Wales Hospital Hong Kong, 82% of PD patients and 87% of HD patients were receiving ESAs, respectively.

In Hong Kong, the most common ESA therapies used are the longer-acting agents, either darbepoetin alfa or methoxy polyethylene glycol-epoetin beta. They are usually given on a monthly basis. The frequency and dosages of ESA therapy are reviewed regularly to achieve the target Hb level as recommended by the KDIGO guidelines. Iron therapy, either in oral or parental form, is also given as required based on patients’ iron status.

### Japan

According to the Japanese Society for Dialysis Therapy Renal Data Registry 2018, the number of long-term dialysis patients in Japan has continued to increase every year, reaching 339,841 at the end of 2018. The number of PD patients was 9,445 in 2018, accounting for 2.77% of the dialysis population.^33^ The percentage of patients undergoing combined PD and HD treatment increased as their duration of PD was lengthened. This is also one of the unique features of PD therapy in Japan. The medical fee of dialysis therapy is fully covered by the public health-insurance system.^34^ Starting in 2018, an extra bonus reimbursement is given to health care facilities if they provide full kidney replacement therapy (KRT) information to patients, treat PD patients at outpatient clinics, or have referred patients to kidney transplantation. This will likely change the overall KRT landscape in Japan in the near future.

In Japan, the target Hb level for PD patients is 11 to 13 g/dL, whereas the target for HD patients is 10 to 12 g/dL.^35^ ESA is administered through the IV route for those on combined therapy, while patients receiving PD alone are given ESAs subcutaneously. Among all dialysis patients, 11.9% do not use ESAs. For patients who use ESAs, the most common ESA used is darbepoetin alfa, followed by epoetin alfa, epoetin beta, epoetin beta pegol, and epoetin kappa (epoetin alfa biosimilar).^36^

### Malaysia

According to the 26th Report of the Malaysian Dialysis and Transplantation Registry, the number of patients receiving dialysis in Malaysia increased from 19,430 in 2008 to 44,136 in 2018.^37^ The increase in the dialysis population was mainly contributed to by the rapid growth in the private HD sector over the past 10 years. Among all dialysis patients in 2018, only 9.9% were receiving PD. By the end of 2018, there were 51 PD centers in Malaysia, with a total of 4,543 PD patients. Most PD centers (36 centers) were operated by the Ministry of Health.

In 2016, a total of 91% of HD patients and 83% of PD patients received ESAs.^38^ Patients dialyzing in government centers are fully covered for the cost of ESA therapy, and since 2009, there has been a national scheme by the Ministry of Health to subsidize ESA therapy for patients in centers run by nongovernment organizations. There is also some reimbursement for ESA therapy under social security funding.

Shorter-acting ESAs are most commonly used and the median weekly ESA dose in HD and PD patients was 5,000 units and 4,500 units per week respectively. In 2016, the median Hb level was 10.2 g/dL in PD patients receiving ESAs, whereas the median Hb level was 11.4 g/dL in those not receiving ESAs. The use of oral iron remained high at 65% of patients receiving PD, while only 15% received parenteral iron. The proportion of PD patients achieving TSATs ≥ 20% was 89%, and 93% achieved target ferritin levels ≥ 100 ng/mL.^38^

### Singapore

The age-standardized incidence rate (ASIR) of dialysis has been consistently higher for HD than PD throughout the years. In 2018, the ASIR was 143.5 per million population and 43.5 per million population for HD and PD, respectively. In 2018, diabetes accounted for 65.8% of new definite dialysis patients, followed by glomerulonephritis (14.1%). Although the ASIR for PD has increased significantly over the years, the ASIR for HD has remained relatively stable. However, the age-standardized prevalence rate for PD has remained relatively stable during this period. Patients who are older and have comorbid medical conditions are preferentially placed on PD, a gentler therapy than HD. As of the end of 2018, almost all prevalent PD patients were managed in the public sector (99.7%), with a minority under the care of voluntary welfare organizations and the private sector. However, most prevalent HD patients were dialyzed in centers run by voluntary welfare organizations (62.8%), followed by the private sector 35.6% and then the public sector (1.6%).^39^

Based on Singapore Renal Registry report number 10 (2012-2013), overall use of ESAs was 92% among the dialysis population, HD use was 92.4%, and PD use was 89.1%. The use of ESAs has remained relatively stable over the years.

In 2018, the percentage of PD patients receiving ESAs was 86.3%, and the proportion of prevalent PD patients who fulfilled the adequate management of anemia criteria of Hb level ≥ 10 g/dL was 66.1%. Among PD patients receiving ESAs, 63.4% achieved Hb levels ≥ 10 g/dL as compared with 83.9% in PD patients not receiving ESAs. The ESAs available in Singapore include shorter-acting recombinant human erythropoietin (rHuEPO) such as epoetin alfa and epoetin beta together with longer-acting ESAs such as darbepoetin alfa and longer-acting erythropoietin receptor activators such as methoxy polyethylene glycol-epoetin beta. The most commonly used ESA is epoetin beta due to its relatively lower cost. The cost of ESAs can be either covered by insurance (MediShield) or subsidized by Medifund, which is a government fund managed by a medical social worker for low-income patients.^39^

### South Korea

The total number of patients on KRT was 103,984 in 2018, and there were 83,867 dialysis patients. Of the dialysis patient population, 77,619 (92.5%) were receiving HD while 6,248 (7.4%) were receiving PD. PD accounts for only 7.4% of the overall dialysis population.^40^ The low PD penetration in South Korea is mainly related to the Medical Insurance System because the reimbursement provides less incentive or even disincentive for PD as compared with HD.

According to the Medical Insurance System guidelines for rHuEPO therapy, the use of ESAs for estimated glomerular filtration rates < 30 mL/min/1.73 m^2^ can be started at Hb levels < 10 g/dL, and Hb levels should be maintained at <11 g/dL. The ESA can be given as rHuEPO 1 to 3 times per week or darbepoetin alfa 2 to 4 times per month or methoxy polyethylene glycol-epoetin beta once per month. In 2018, mean Hb levels for PD and HD patients were 10.3 and 10.4 g/dL, respectively, and 84% of PD patients were using ESAs. Most patients used darbepoetin alfa or methoxy polyethylene glycol-epoetin beta.^40^

The guidelines for IV iron therapy by the Medical Insurance System stipulate that IV iron can be given to HD patients if serum ferritin level is <200 ng/mL or TSAT is <20% or to PD patients if an oral iron application is not feasible or adequate as supplement and serum ferritin level is <100 ng/mL or TSAT is <20%. For erythropoietin-resistant patients receiving dialysis, target iron status is modified up to serum ferritin levels > 300 ng/mL or TSATs > 30%.

### Thailand

The Thai government provides universal health care to all Thai citizens through 3 health insurance schemes. One of them is the universal coverage scheme, which covers >70% of the Thai population who are self-employed and do not work as government officials, as well as state enterprise and private establishment employees.^41^ Initially, this scheme only offered basic and comprehensive health care while KRT was not included due to its high cost. The PD-first policy was implemented in Thailand in 2008 as a model of initial treatment of patients with ESKD under the universal coverage scheme. As of 2016, there were 26,450 active PD patients in 231 PD centers in 13 health care regions in Thailand, which has increased 10-fold during the past decade, while more than 150 new PD centers have been operated in the last decade. PD penetration in 2016 was 31% compared with <10% before 2008.^41^^,^^42^

The mean Hb level of PD patients in Thailand was 10.2 ± 1.7 g/dL, and 41.8% of Thai PD patients had an Hb level < 10 g/dL. One of the main reasons for this is the limitation of ESA dosage reimbursement by the policy. Most Thai PD patients were receiving shorter-acting ESAs, which are lower cost, with 92.8% of them using epoetin alfa. The mean ESA dose used was 4,982 ± 3,168 IU/wk, which is relatively low compared with other countries.^43^ The prevalence of anemia (Hb < 10 g/dL) tended to increase with higher serum ferritin level. The 52.3% of PD patients whose serum ferritin levels were between 801 and 1,200 ng/mL were anemic compared with 25.9% of PD patients whose serum ferritin levels were <200 ng/mL. This may be due to ineffective iron use because of inflammation. IV iron therapy was used in only 8% of Thai PD patients. Younger age (aged <45 years), lower serum albumin level, and higher serum ferritin level (serum ferritin > 200 ng/mL) were independent risk factors for anemia.^44^^,^^45^

Table 1 compares the percentage of prevalent patients on PD in the Asia Pacific region. Table 2 shows the comparison of use of ESA among PD patients in Asia Pacific areas.

**Table 1:** Comparison of Percentage of Prevalent Dialysis Patients Receiving PD in Asia Pacific Region

	No. of Prevalent Dialysis Patients	Percentage of Dialysis Patients on PD	Year	Reference
China	610,881	14.1%	2017	25
Hong Kong	6,094	74.6%	2018	30
Japan	339,841	2.8%	2018	32
Malaysia	44,136	9.9%	2018	36
Singapore	7,405	13.7%	2018	38
South Korea	83,867	7.4%	2018	39
Thailand	94,406	31%	2016	40

Abbreviation: PD, peritoneal dialysis.

**Table 2:** Use of ESAs Among Patients Receiving PD in the Asia Pacific Region

	Proportion of PD Patients on ESA	Most Commonly Used ESA	Year	Reference
China	89%	Shorter -acting ESA	2018	27
Hong Kong	82.0%	Darbepoetin alfa or methoxy polyethylene glycol-epoetin beta	2018	30
Japan	NA	Darbepoetin alfa	2014	35
Malaysia	83%	Shorter-acting ESA	2016	37
Singapore	86.3%	Epoetin beta	2018	38
South Korea	84%	Darbepoetin alfa or methoxy polyethylene glycol-epoetin beta	2018	39
Thailand	96%	Epoetin alfa	2017	40, 41

Abbreviations: ESA, erythropoiesis-stimulating agent; NA, not available; PD, peritoneal dialysis.

## Discussion

Anemia is a well-recognized complication of CKD. The number of patients with ESKD is growing rapidly in Asia as well as globally, which poses a great financial burden to many countries. Iron supplements and ESAs remain the cornerstone of anemia treatment. However, the overall penetration of PD and use of ESAs are greatly affected by individual governments’ health care reimbursement policies.

A recent study of the French Language Peritoneal Dialysis Registry from 2010 to 2017 found that of 568 PD patients, 74% were treated with ESAs, 23% were receiving oral iron, and only 11% had received IV iron.^46^ The average Hb level achieved was close to 12 g/dL. In our Asia Pacific region, 82% to 96% of PD patients are receiving ESAs (Table 2).

Asia is diversified, with different ethnic groups, cultures, and medical practices. Common clinical practice guidelines specific to Asian countries and regions are important because they take into account the diversified socioeconomic structures. The emergence of new drug treatments for anemia including HIF-PHIs will also change the treatment possibilities. There are not many large-scale randomized controlled trials on the use of iron in PD patients, and the optimal route and dosage of iron administration in PD requires more research.

With a gradually increasing number of dialysis patients in the Asian Pacific region, establishing a kidney registry across the region may be useful for comparison of epidemiologic data and clinical practice to improve anemia management in the PD population.
